# Transcatheter Aortic Valve Replacement and the Mick Jagger Phenomenon

**DOI:** 10.1016/j.shj.2023.100233

**Published:** 2023-11-15

**Authors:** Anthony DeMaria

**Affiliations:** Judy and Jack White Chair in Cardiology, Sulpizio Cardiovascular Center, University of California, San Diego, La Jolla, California, USA

Considerable evidence exists that heart valve disease often is unrecognized and, even when diagnosed, is undertreated. The OxValve study found that 5% of randomly surveyed Europeans had evidence of moderate to severe valve disease.^1^ Another study found that many elderly patients were not referred for intervention even when meeting guideline criteria for such procedures.^2^ Accordingly, there exists a real need for a greater awareness of the prevalence and availability of therapy for valve disease among both patients and physicians. The ability to treat valve disorders percutaneously has only amplified the need for such awareness.

Few celebrities are more recognizable and famous than Mick Jagger, the lead singer of the Rolling Stones, even among those as chronologically gifted as me. He is a consummate showman, dancing about the stage during his performances exerting energy equivalent to many structured cardio exercise workouts. So, it was big news and well reported in the lay press when he underwent a transcatheter aortic valve replacement (TAVR) procedure for aortic stenosis in April 2019. It was important that he was able to perform so well up to the point of his procedure even with significant heart disease. In fact, he had to abruptly cancel a tour that was about to begin. Perhaps even more noteworthy was that he was out of the hospital in days and was able to return to rehearsal and touring just weeks after the TAVR. The existence of this nonsurgical procedure to treat serious aortic stenosis was widely covered in both print and video media. Although protecting the privacy of his health and limiting specifics, Mr. Jagger’s illness did more to inform the public, and perhaps some in medicine, of the existence of relatively asymptomatic valvular disease and the new nonsurgical approaches available for its treatment than most other efforts to communicate this information.

There are a number of interesting aspects to the reaction to and effect of Mick Jagger’s TAVR. I am certain that a number of well-recognized individuals have now undergone TAVR. In fact, I am aware that Henry Kissinger, a statesman of enormous international reputation, underwent the procedure. Nevertheless, even an extensive Internet search did not yield notice of individuals other than Jagger whose TAVRs were well publicized. He is clearly a very colorful individual who marches to his own drummer and whose band exemplifies the rebellion that is often experienced by youth. In addition, his performances demand an enormous degree of exertion. So, his return to performing a vigorous program shortly after having a “new heart valve” was sure to attract attention. This would be analogous to an elite athlete undergoing a TAVR in midseason and rapidly returning to competition thereafter. Regardless of the reason, it is interesting that Mick Jagger’s TAVR attracted such a disproportionate degree of attention.

I observed an interesting pattern of reporting in the media articles describing Jagger’s diagnosis and treatment. The fact of his diagnosis and TAVR would appear at the beginning of the article or video clip. Then a physician, most often cardiologist or cardiac surgeon, would be interviewed as to generally what aortic stenosis was and how it was treated. Most importantly, they usually focused on how it formerly required open-heart surgery but could now be treated percutaneously. In terms of dissemination of information, or “spreading the word,” it would be hard to envision a better venue. It was also of value for the individual medical expert who provided the descriptions. So, Jagger’s illness was of great value for society, the medical profession, and individual experts.

The education of society about medical disorders in conjunction with the illness of celebrities is not new. The pharmaceutical and device industries do, of course, carry out substantial advertising to the public. Often this involves a celebrity who relates aspects of their symptoms, diagnosis, and treatment. Also, individual celebrities frequently come forward and divulge their own experiences with illness in an attempt to inform and help others dealing with the same problems. This often occurs in conjunction with psychiatric problems. There is no question that this is a very effective way to disseminate information. It is much less common for the illness of a celebrity to become public and informational independent of their own efforts or of the medical industry. The coronary artery disease of former President Bill Clinton comes to mind. Mick Jagger’s aortic stenosis is certainly in this category.

The field of structural heart disease is one of the most, if not the most, rapidly evolving and advancing areas of cardiovascular medicine. It seems like new effective diagnostic and therapeutic procedures are being developed monthly. This explosion of technology carries with it a proportional need to inform and educate not only our colleagues but the public about these advances. This need is evidenced by the prevalence and undertreatment of heart valve disease that currently exists. Obviously the “phenomenon” surrounding Mick Jagger’s aortic stenosis and TAVR resulted in the dissemination of an enormous amount of information to both physicians and society in general. It raises the question of whether we as physicians should discuss with our very famous patients the possible benefit they could convey in making their illness and treatment public.

## Funding

The author has no funding to report.

## Disclosure Statement

The author reports no conflict of interest.

## References

[1] d'Arcy J.L., Coffey S., Loudon M.A. (2016). Large-scale community echocardiographic screening reveals a major burden of undiagnosed valvular heart disease in older people: the OxVALVE Population Cohort Study. Eur Heart J.

[2] lung B., Cachier A., Baron G. (2005). Decision-making in elderly patients with severe aortic stenosis: why are so many denied surgery?. Eur Heart J.

